# Control of *Aedes* mosquito populations using recombinant microalgae expressing short hairpin RNAs and their effect on plankton

**DOI:** 10.1371/journal.pntd.0011109

**Published:** 2023-01-26

**Authors:** Xiaowen Fei, Sha Xiao, Xiaodan Huang, Zhijie Li, Xinghan Li, Changhao He, Yajun Li, Xiuxia Zhang, Xiaodong Deng

**Affiliations:** 1 Department of Biochemistry and Molecular Biology, Hainan Medical University, Haikou, China; 2 Institute of Tropical Bioscience and Biotechnology, Chinese Academy of Tropical Agricultural Science & Key Laboratory of Biology and Genetic Resources of Tropical Crops of Hainan Province, Hainan Institute for Tropical Agricultural Resources, Haikou, China; 3 Hainan Provincial Key Laboratory for Functional Components Research and Utilization of Marine Bio-resources, Haikou, China; 4 Zhanjiang Experimental Station, CATAS, Zhanjiang, China; Connecticut Agricultural Experiment Station, UNITED STATES

## Abstract

New biocontrol strategies are urgently needed to combat vector-borne infectious diseases. This study presents a low-cost method to produce a potential mosquito insecticide that utilizes the microalgae released into suburban water sources to control mosquito populations. *Chlorella* microalgae are ubiquitous in local waters, which were chosen as the host for genetic transfection. This species facilitated the recombinant algae to adapt to the prevailing environmental conditions with rapid growth and high relative abundance. The procedure involved microalgae RNAi-based insecticides developed using short hairpin RNAs targeting the *Aedes aegypti* chitin synthase A (*chsa*) gene in *Chlorella*. These insecticides effectively silenced the *chsa* gene, inhibiting *Aedes* metamorphosis in the laboratory and simulated-field trials. This study explored the impact of recombinant microalgae on the phytoplankton and zooplankton in suburban waters. High-throughput sequencing revealed that rapid reproduction of recombinant *Chlorella* indirectly caused the disappearance of some phytoplankton and reduced the protozoan species. This study demonstrated that a recombinant microalgae-based insecticide could effectively reduce the population of *Aedes* mosquitoes in the laboratory and simulated field trials. However, the impact of this technology on the environment and ecology requires further investigation.

## Author summary

A new low-cost strategy was developed to control vector mosquitoes safely. *Chlorella* was genetically modified to produce siRNA targeting the mosquito *chsa* gene, inhibiting gene expression after the larval stage. The inhibition prevented metamorphosis with high larval mortality in both *Aedes aegypti* and *Aedes albopictus*. The recombinant *Chlorella* performed well in the simulated field trials but impacted plankton populations in the released water, which indicates that the technology requires further optimization for field use. This approach is promising for biorational mosquito control to prevent mosquito-borne diseases.

## Introduction

*Aedes* spp. is important for transmitting several mosquito-borne diseases. *Aedes aegypti* and *Aedes albopictus* are the primary carriers of pathogens, such as dengue, Zika, yellow fever, and chikungunya viruses, thus impacting public health [1,2]. Approximately half of the world’s population is infected with these mosquito-borne diseases annually, with 96 million manifesting as clinical diseases with varying severity levels [3]. In China, the incidence of dengue fever was low between 2000 and 2011 and has increased rapidly since then. A 2014 outbreak of dengue fever involved 46,864 reported cases. Between 2015 to 2018, the incidence varied with a clear upward trend in 2019 (22,599 reported cases) [4]. Another disease, the Zika virus (a member of the *Flaviviridae* family), is associated with neonatal microcephaly and Guillain-Barré syndrome. Chikungunya virus, a member of the *Togaviridae* family, causes a febrile illness characterized by severe chronic arthralgia [5,6].

Many *Aedes* mosquitoes live in or near human dwellings, and their life cycle involves four stages: egg, larva, pupa, and adult. When the egg is exposed to water, it hatches larvae within a few hours, develops into fourth instars within 4–7 days, and enters the pupal stage. The pupa undergoes metamorphosis, molting into the winged adult form. At this time, the females are reproductively mature and are capable of transmitting pathogens while feeding on human hosts. Unfortunately, mosquitoes are developing resistance to insecticides very fast and produce numerous offspring, generating more populations with different genetic characteristics and worsening insect resistance to insecticides [7,8]. Alternative vector control strategies, such as predatory fish and fungi, have been developed to control vector populations [9]. *Bacillus thuringiensis* toxin and *Wolbachia* infection can effectively control mosquitoes [10–13]. However, their effectiveness requires an in-depth assessment for long-term use.

In this study, the mosquito chitin synthase A (chsa) gene was the RNA interference (RNAi) target for controlling mosquito multiplication. Chitin, an N-acetylglucosamine homopolymer composed of N-acetyl-D-glucosamine-[GlcNAc] linked by beta-1,4-glycosidic bonds, is a major structural component of the exoskeleton cuticle and an important component of the peritrophic matrix (PM) in the mosquito intestine and eggshell [14–16]. Chitin synthase (CHS), a member of the glycosyl transferase family, completes the final step of chitin biosynthetic. Insects usually have two chitin synthase genes: *chsa* and *chsb*. *Chsa* mainly synthesizes chitin in the exoskeleton cuticle and eggs, while *chsb* synthesizes chitin in the intestinal PM [17–19]. As chitin is crucial for mosquito life stages, it is an attractive target for controlling mosquito development and life cycle. Chitin, an important structural component of invertebrates, is absent in vertebrates, making it an attractive insect-specific target with low vertebrate toxicity.

In this study, RNAi technology was widely used to examine gene function in numerous insect species and has been explored as an alternative for insect population control [20–23]. In target cells, double standard RNA (dsRNA) is cleaved by RNase III-dicer into small interfering RNA (siRNA) of 20–25 nucleotides in length. Subsequently, the argonaute protein assembles with the siRNA to form an RNA-induced silencing complex (RISC), which binds to and degrades complementary endogenous messenger RNA (mRNA). Currently, dsRNA administration methods include immersion, oral feeding, and microinjection [24–28]. The specificity of RNAi makes these methods environmentally safer than other approaches currently, minimizing toxicity to non-target species and reducing the likelihood of resistance in insect populations [29–31].

In the current study, a low-cost mosquito insecticide alternative utilizes transgenic microalgae to control mosquito populations in suburban waters. Since chitin mainly exists in fungi and invertebrates, it has minimal effects on vertebrates. So, it is considered a safe target for human health and safety. This study also explores the impact of recombinant microalgae on phytoplankton and zooplankton populations in suburban waters via high-throughput sequencing, laying a foundation for the safe use and evaluation of recombinant microalgae.

## Methods

### Mosquito maintenance

The rearing of mosquitoes was performed as previously reported [32,33]. *Ae*. *aegypti* Rockefeller strain mosquitoes were donated by Professor Han Qian of Hainan University. The local wild type *Ae*. *albopictus* were captured in Haikou, China, and propagated for 5 generations in the laboratory. Both mosquito species were kept in an insectary at the Chinese Academy of Tropical Agricultural Science. The mosquitoes were maintained at 26°C and 70–80% relative humidity.

Additionally, the mosquito cages contained a sponge soaked with sugar water, and chicken blood was used as the blood meal for female mosquitoes. After the female mosquitoes laid eggs on wet filter paper, the eggs were collected and kept dry. The eggs were then placed in incubation water (containing rat food) for hatching.

### Microalgae strains and plasmids

The *Chlorella vulgaris* HOC5 strain was isolated from local waters in Hainan, China, and cultured in BG11 medium. Liquid cultures were maintained at 25°C in a shaker (200 rpm) under continuous light [34]. Next, plasmid pMD18-T was locally constructed by our research group and kept in our laboratory. The pMaa7 IR / XIR RNAi expression vector was purchased from the *Chlamydomonas* center (Duke University, Durham, NC, USA).

### Construction of RNAi vector pMaa7 IR/CHSAIR

Complementary DNA (cDNA) of *Ae*. *aegypti* was used as the polymerase chain reaction (PCR) amplification template. The 331 to 621 region of the *chsa* gene (AAEL002718) was PCR-amplified using two primers CHSAF(5’-aaatcctgaa aatcttcgcc- 3’) and CHSAR (5’-cttgaatggtttcttcatcg-3’). The amplified fragment was inserted into the pMD18-T plasmid backbone (pMD-CHSA). The fragments were digested with HindIII/BamHI and XbaI/SalI and inserted into the corresponding cloning sites of plasmid pT282 (pT282-CHSA), which contained the inverted repeat sequence of the *chsa* gene. Both the RNAi (pMaa7 IR/XIR) and intermediate (pT282-CHSA) vectors were digested using EcoRI and then ligated to produce the RNAi recombinant pMaa7 IR/CHSAIR [35].

### Microalgae transfection

The *Chlorella* was grown and collected by centrifugation and resuspended in an electroporation buffer. For transfection, 2 μg of pMaa7IR/CHSAIR was mixed with the recipient *Chlorella* and incubated on ice for 15 min. The mixtures were then transferred into an electroporation cuvette and subjected to an electric pulse in an electrometer (BTX, ECM 630, USA) with a 1600 V/cm voltage and a pulse time of 1 ms. Then, the suspension was transferred to BG11 medium containing 60 mmol/L sorbitol and allowed to recover overnight. Finally, the cells were collected by centrifugation (10000 g, 3 min) and plated on BG11 agar plates containing 10 μg/mL paromomycin until the algal colonies appeared after 5–10 days [36,37].

### Mosquito feeding experiments

After the RNAi vector, pMaa7 IR/CHSAIR had been transferred into *Chlorella*. The transgenic microalgae were characterized using PCR to determine if the *chsa* inverted repeat expression cassette had been integrated into the *Chlorella* genome. The positive clones were used to feed the mosquitoes in the laboratory. As previously described, ten and 300 mosquito feeding experiments were performed in the insectary [33]. In the 10 mosquito feeding experiment, The mosquitoes were divided into experimental and control groups. Each group contained 10 L1 larvae in 5 mL water supplemented with 5 mg fresh microalgae. The larvae in the experimental groups were fed the *chsa* RNAi transgenic *Chlorella* strains (CHSA1-5), respectively. In contrast, the larvae in the control groups were fed wild-type *Chlorella HOC5*, water, fodder, and the empty plasmid pMaa7IR/XIR transgenic strain. The experiments were performed in triplicate, recording the mortality, pupation, and adult emergence rates.

In the 300 mosquito feeding experiment, 300 L1 larvae were placed into 50 mL of water supplemented with 20 mg of fresh microalgae. The larvae feeding the recombinant *Chlorella* CHSA5 were set as the test treatment, whereas the larvae fed with *Chlorella HOC5*, water, and fodder were used as controls. The triplicate experiments included records of mortality, pupation, and adult emergence rates.

### Verification of mRNA knockdown in *Aedes* larvae

For quantitative real-time polymerase chain reaction (qRT-PCR), 10–20 L3 larvae were collected and pooled from each treatment. Total RNA was isolated from the larvae using the TRIzol Reagent (Takara, Shiga, Japan), following the manufacturer’s instructions. Single-strand cDNA was synthesized from total RNA using oligo-dT primers (Shenggong, Shanghai, China). Real-time PCR was performed on a BioRad iCycler iQ Real-Time PCR Detection System (BioRad, Hercules, CA, USA) using SYBR green as a fluorescent dye. Primers targeting the *Aedes* RPS17 gene (Forward: 5’-AAGAAGTGGCCATCATTCCA-3’ and Reverse: 5’-GGTCTCCGGGTCGAC TTC-3’) were used as internal controls. The *chsa* primers were CHSAF (5’-aaatcctgaaaatcttcgcc- 3’) and CHSAR (5’-cttgaatggtttcttcatcg-3’). The amplification rate of each transcript (Ct) was calculated using the PCR baseline subtraction method using the iCycler software (constant fluorescence level). The cycle threshold (Ct) was determined in triplicate, and the relative fold differences were calculated using the relative quantification method (2^-ΔΔCT^) [38]. *Chsa* expression was determined relative to the endogenous control, *Ae*. *aegypti* RPS17 (Ribo ribosomal protein S17, GenBank accession no. AAEL004175 (KY000705)) [39].

### Water quality and *Chlorella* growth in the target water area

Water samples (10 L) were collected from the Meishe river, Shapo reservoir, and Hongcheng lake in Haikou City, Hainan, China. A model DR3900 Laboratory Spectrophotometer (HACH, CO, USA) was used to measure the concentrations of nitrogen, phosphorus, ammonia nitrogen, nitrite nitrogen, chemical oxygen demand (COD), and silicate. After centrifuging the water samples at 5000 rpm for 5 min, the algal species were observed and identified by light microscopy. Subsequently, 30 mL of 2x10^7^ *Chlorella* HOC5 was inoculated into the 10 L water samples, and the *Chlorella* growth kinetics were observed and recorded.

### Simulated-field trials

Simulated field trials were performed following the Mysore *et al*. with modification using 1.5 m^3^ volume and 0.4 mm aperture polyester mesh breeding mosquito cages [40]. Four 1000 L buckets for microalgae culture were placed in cages with LED lights to facilitate algal photosynthesis in water. A ventilation pump was used to ensure the continuous circulation of the water. Then, 10 L of the cultured microalgae was poured into the buckets. Aeration and sufficient light were maintained for the culture of *Chlorella* from 10 L to 200 L. Then, Meishe river water was added to adjust the algal volume to 800 L at 2x10^6^-2x10^7^ cells / mL. The aeration pump pressure was adjusted to make the water flow in the barrel. Approximately 1000 L1 larvae were placed in each cage. A 10% sucrose solution was prepared for male adults and chicken blood for egg-laying females. The number of adult *Aedes* mosquitoes was counted weekly similar to the abundance of *Chlorella* determined in the water. One day before mosquito counting, the blood meal and sugar water were discontinued (to reduce the physical strength of mosquitoes temporarily). The counting period was between 3:00 and 6:00 a.m. When counting, a dim LED flashlight was used to illuminate the mosquito cages.

### Sample preparation and DNA extraction of test water

An 18S high-throughput sequencing analysis was performed on the water from the simulated-field trials to detect the effect of *Chlorella* on the phytoplankton and zooplankton in the test water. After feeding *Aedes* for 28 days, a 1 L water sample was collected from the *Aedes*-containing bucket and filtered through a 0.40 μm polycarbonate membrane (Millipore, Burlington, MA, USA) under a 30 kPa vacuum to collect the plankton. The membranes were stored at −80°C until the time of analysis. Genomic DNA (gDNA) was extracted using a modified Winnepenninckx et al. method [41].

### Amplification and sequencing of the V4 hypervariable region

The V4 hypervariable region of the 18S rDNA was selected as the target for high-throughput sequencing analysis. The targeted region was amplified using a pair of eukaryotic universal primers (D514, 5′-TCCAGCTCCAATAGCGTA-3′, and B706R, 5′-AATCCRAGAATTTCACCTCT-3′) [42,43]. Sequence libraries were constructed using the NEB Next Ultra DNA Library Prep Kit for Illumina (Illumina, CA, USA) as described in the manufacturer’s protocol. The library quality was assessed using the Qubit Fluorometer and Agilent Bioanalyzer 2100 system (Agilent, CA, USA). Finally, the libraries were sequenced using an Illumina HiSeq2500 platform. Low-quality reads were filtered to generate high-quality clean tags using QIIME (Version 1.7.0). Singletons were removed from the dataset, and sequences with > 97% similarity were clustered into operational taxonomic units (OTUs) using UPARSE (Version 7.0.1001) [44,45]. Assignments of the taxonomic OTUs were completed using the Silva and Nucleotide Databases in National Center for Biotechnology Information (NCBI) (https://blast.ncbi.nlm.nih.gov) [46]. Sample diversity was determined using QIIME (Version 1.7.0), and Shannon indexes were calculated following the Whittaker [47] protocol.

### Statistical analyses

Data analysis was performed using Statistical Package for the Social Sciences (SPSS) and presented as mean ± S.D. (standard deviation). Duncan’s multiple ranges and *t*-test tests were performed to examine significant differences between means. Values with p-value < 0.05 were considered statistically significant. Asterisks indicate the statistical significance in all cases: *P <0.05, **P <0.01. Error bars show the standard deviation.

## Results

### chsa RNAi transgenic *Chlorella* are lethal to *Ae*. *aegypti*

**Fig 1:**
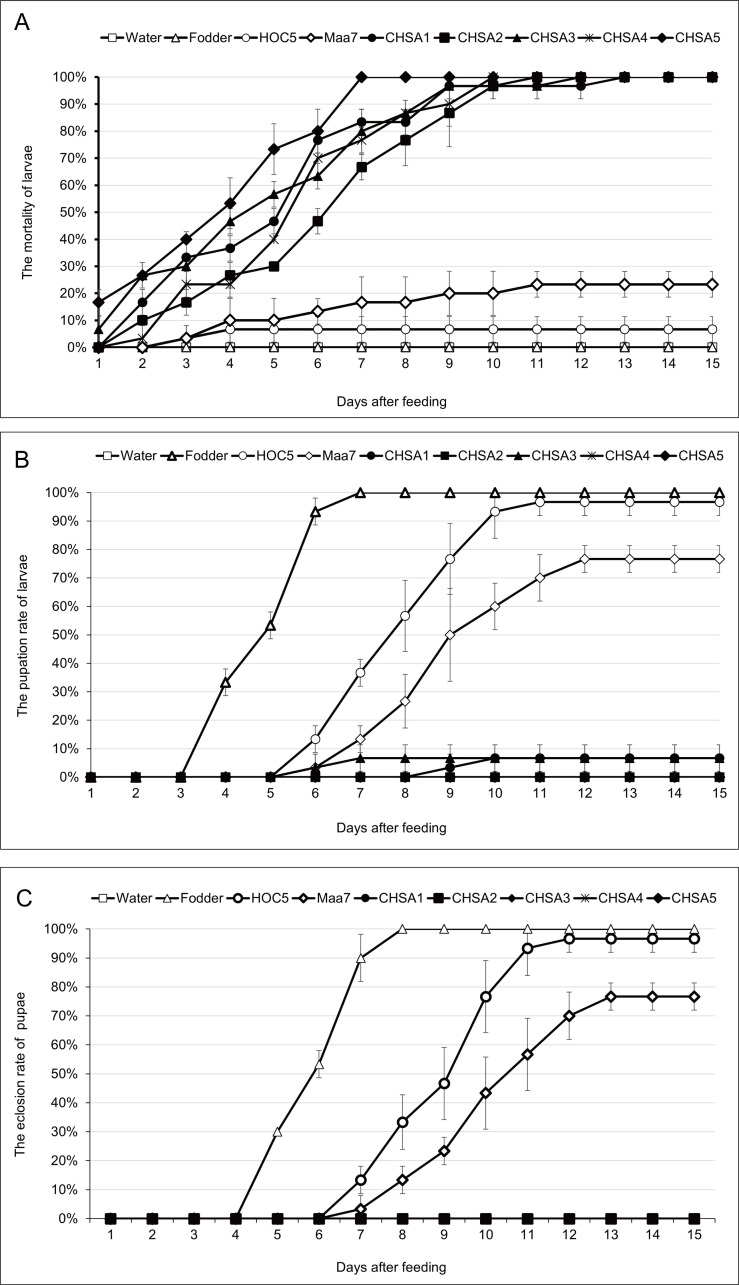
Mortality (A), pupation (B), and eclosion rates (C) of *Aedes aegypti* fed with *chsa* RNAi recombinant *Chlorella*. Water: larvae fed with water; Fodder: larvae fed with fodder; HOC5: larvae fed with wild *Chlorella vulgaris* HOC5; Maa7: larvae fed with empty plasmid Maa7IR/XIR transgenic *Chlorella* strain; CHSA1 to CHSA5: larvae fed with Maa7IR/CHSAIR transgenic *Chlorella* strains CHSA1 to CHSA5. Each treated or control group contained ten *Aedes* larvae, and the experiments were performed in triplicate for 15 days.

The RNAi target gene is the *chsa* gene (AAEL002718) of *Ae*. *aegypti*. The target region for silencing by the RNAi was nucleotides 331 to 621 in the CDS. cDNA of *Ae*. *aegypti* was used as a template to amplify the *chsa* RNAi interference fragment, and an amplicon of approximately 290 bp was obtained. This fragment was then cloned into pMaa7IR/XIR in forward and reverse directions to produce the recombinant RNAi vector of pMaa7IR/CHSAIR. After the RNAi vector was transformed into *C*. *vulgaris HOC5*, 79 positive recombinant algal clones were identified by PCR, and five were used for subsequent experiments. In the 10 mosquito feeding experiment, the larvae fed recombinant *Chlorella* clones CHSA3 and CHSA5 began to die on the second day. Larvae fed clones CHSA1, CHSA2 and CHSA4 began to die on the third day and all the larvae fed recombinant *Chlorella* died within 13 days. None of the larvae died when fed with water and fodder, but only 6.7 and 23.3% died within 15 days after feeding on *Chlorella HOC5* and empty-plasmid Maa7IR/XIR transgenic *Chlorella*. Orally feeding *Aedes* larvae with *chsa* RNAi recombinant *Chlorella* is lethal (Fig 1A).

Only 6.67% of *Aedes* mosquito larvae fed recombinant *Chlorella* CHSA1 and CHSA3 pupated, and none pupated after feeding on recombinant *Chlorella* CHSA2, CHSA4, and CHSA5. In the control groups, 100% of the larvae pupated when fed on fodder. 96.7 and 76.7% of pupation rates were observed in larvae fed on *Chlorella HOC5* and empty plasmid Maa7IR/XIR transgenic *Chlorella*, respectively. However, no larva pupated when fed on water alone (Fig 1B).

None of the pupae developed into adults after feeding on any of the recombinant *Chlorella* strains. In contrast, 100, 96.67, and 76.67% of control pupae emerged into adults when fed on fodder, *Chlorella HOC5*, and empty plasmid Maa7IR/XIR transgenic *Chlorella*, respectively (Fig 1C).

### Feeding experiment of 300 *Ae*. *aegypti* mosquitoes

**Fig 2:**
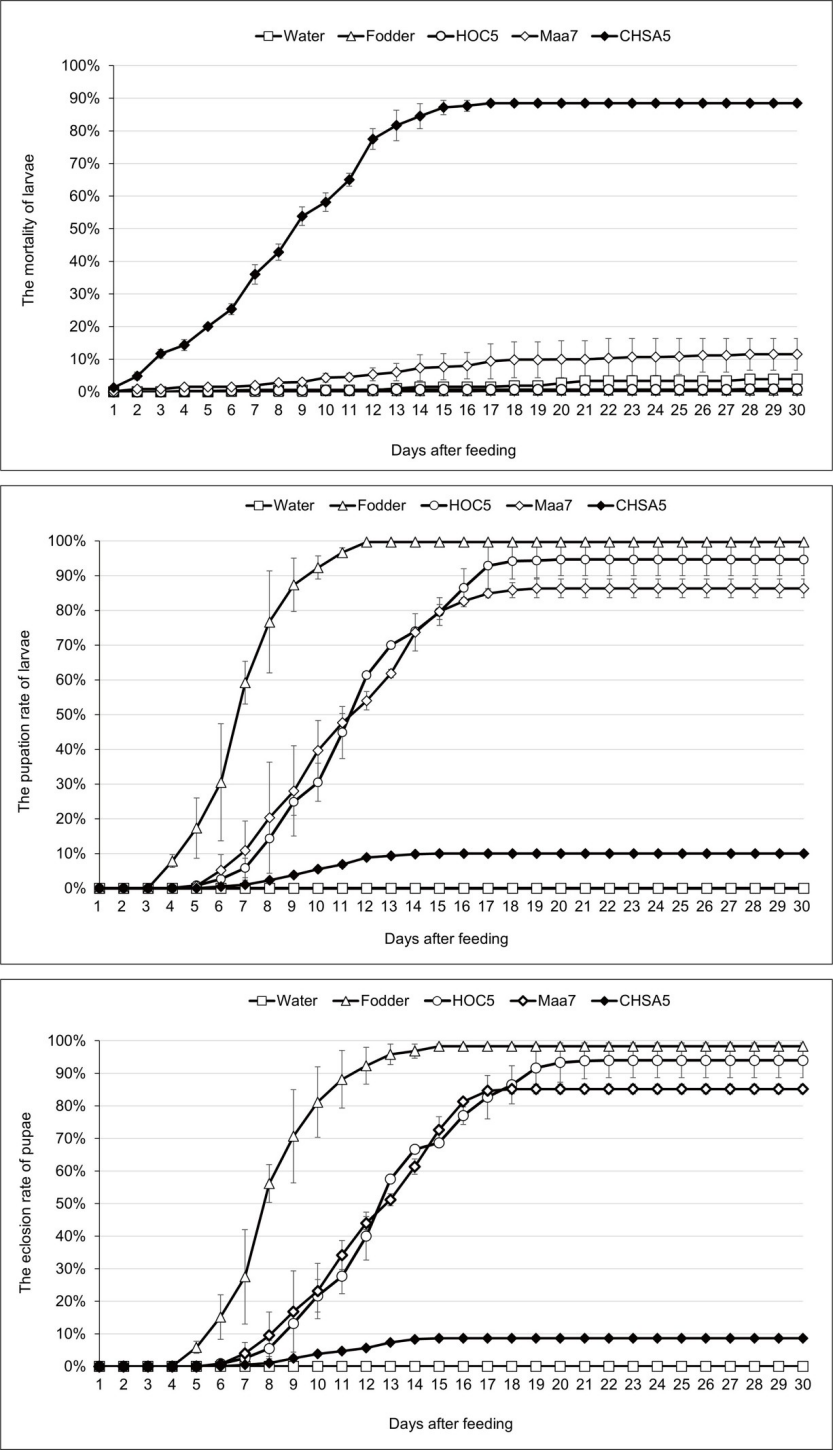
Mortality (A), pupation (B), and eclosion rates (C) of *Aedes aegypti* fed with *chsa* RNAi recombinant *Chlorella*. Water: larvae fed with water; Fodder: larvae fed with fodder; HOC5: larvae fed with wild *Chlorella vulgaris* HOC5; Maa7: larvae fed with empty plasmid Maa7IR/XIR transgenic *Chlorella* strain; CHSA5: larvae fed with recombinant *Chlorella* strain CHSA5. Each treated or control group contained 300 *Aedes* larvae, and the experiments were performed in triplicate for 30 days.

Approximately 300 L1 *Ae*. *Aegypti* larvae in each treatment were assessed using feeding experiments for 30 days. The larvae fed recombinant *Chlorella* CHSA5 began to die on the second day, and 88.5% of larvae died within 30 days. In the controls, only 3.8, 0.3, 0.8, and 10.3% of the larvae died after feeding on water, fodder, *Chlorella HOC5*, and Maa7IR/XIR transgenic *Chlorella*, respectively (Fig 2A).

The larvae fed on fodder started pupating on day 4 and 99.7% of larvae pupated by day 12. The larvae fed *Chlorella HOC5* started pupating on day 5, and by day 20, 94.7% pupated. Besides, the larvae fed on Maa7IR/XIR transgenic *Chlorella* began to pupate on day 5, and 86.3% pupated by day 19. In contrast, larvae fed on recombinant *Chlorella* CHSA5 began to pupate on day 6, and by day 15, 10.0% pupated (Fig 2B).

The fodder-fed *Aedes* fully emerged into adults on day 15, and 98.3% of the pupae emerged within 30 days. The *Aedes* fed *Chlorella HOC5* fully emerged into adults by day 22, with 94.0% of the pupae emerging into adults. However, only 8.7% of *Aedes* pupae emerged into adults when fed on recombinant *Chlorella* CHSA5 (Fig 2C).

### *Ae*. *albopictus* mosquito feeding experiment

**Fig 3:**
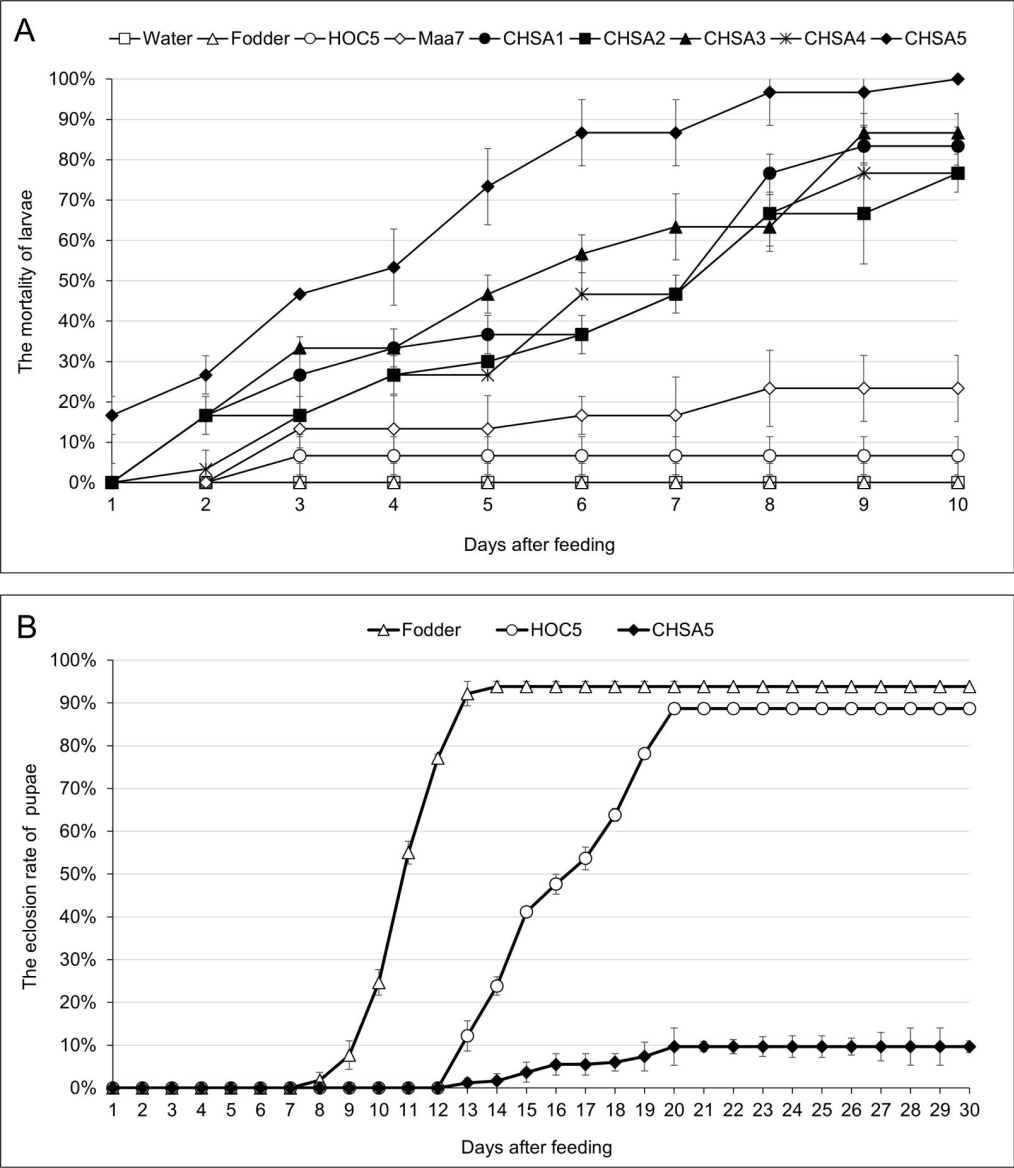
Mortality (A) and eclosion rates (B) of *Ae*. *albopictus* fed with *chsa* RNAi recombinant *Chlorella*. Water: larvae fed with water; Fodder: larvae fed with fodder; HOC5: larvae fed with wild *Chlorella vulgaris* HOC5; Maa7: larvae fed with empty plasmid Maa7IR/XIR transgenic *Chlorella* strain; CHSA1 to CHSA5: larvae fed with recombinant *Chlorella* strains CHSA1 to CHSA5. Each treated or control group contained 10 and 300 *Aedes* larvae in triplicate experiments A and B, respectively.

Similar experiments to those described above were conducted on *Ae*. *Albopictus* mosquitoes. Larvae fed on recombinant *Chlorella* CHSA5 had the highest mortality (100%) on the 10^th^ day, followed by 86.7 and 83.3% mortality rates in larvae fed on recombinant *Chlorella* CHSA3 and CHSA1, respectively. None of the larvae died when fed on water and fodder. Only 6.7 and 23.3% of the larvae died when fed on control *Chlorella* HOC5 or Maa7IR/XIR transgenic *Chlorella*, respectively (Fig 3A). In the 300 *Ae*. *albopictus* mosquito feeding experiment, 9.7% *Aedes* pupae emerged into adults when fed on recombinant *Chlorella* CHSA5. However, 93.8 and 88.7% of the control pupae emerged into adults within 30 days of feeding on fodder and *Chlorella HOC5*, respectively (Fig 3B). These results show that recombinant *Chlorella* is more lethal and effective on *Ae*. *aegypti* than *Ae*. *albopictus* larvae.

### Expression of *chsa* mRNA in *Aedes* larvae fed recombinant *Chlorella*

**Fig 4:**
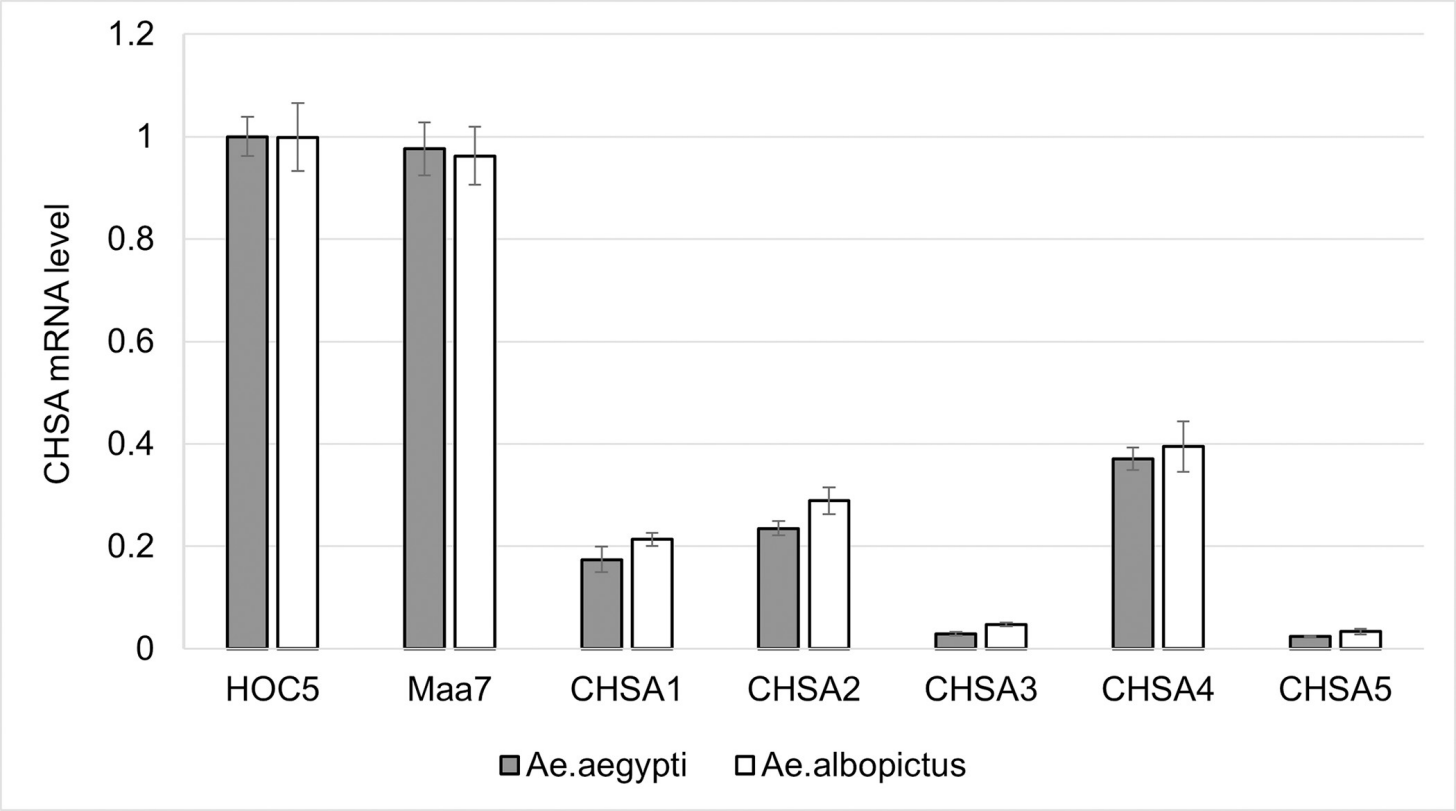
The relative *chsa* mRNA levels in *Aedes* L3 larvae fed with recombinant Chlorella. HOC5: larvae fed with wild *Chlorella* strain HOC5; Maa7: larvae fed with empty plasmid Maa7IR/XIR transgenic *Chlorella* strain; CHSA1 to CHSA5: larvae fed with recombinant *Chlorella* strains CHSA1 to CHSA5.

Real-time PCR was used to examine the expression of the *chsa* gene in *Aedes* larvae fed on recombinant *Chlorella* strains using *Ae*. *Aegypti* larvae fed with *Chlorella HOC5* as controls. The expression of *chsa* gene was significantly lower in *Ae*. *Aegypti* larvae fed on recombinant *Chlorella* than in the control larvae (Fig 4). The lowest rate (97.6%) was observed in larvae fed on clone CHSA5. The *chsa* gene expression was 97.1–62.9% in other larvae fed on CHSA3, CHSA1, CHSA2, and CHSA4 strains. Similar results were obtained using qRT-PCR on *Ae*. *albopictus* fed with the recombinant *Chlorella*. These results suggest that recombinant *Chlorella* can remain effectively silent in the *chsa* gene in *Aedes* mosquitoes.

### Simulated-field evaluation of recombinant *Chlorella* CHSA5 activity

The recombinant *Chlorella* CHSA5 activity was assessed under simulated-field conditions in preparation for future field studies. First, the water quality and local dominant algal strains were tested in three areas of Haikou City to detect whether *Chlorella* CHSA5 is suitable for growth in the above waters. The nitrogen, phosphorus, ammonia, nitrate, nitrite, and chemical oxygen demand (COD) levels in the Meishe river, Shapo reservoir, and Hongcheng lake exceeded the recommended limits of a clean water source. Pollutants and eutrophication were observed in the water bodies. Moreover, the salinity of Hongcheng lake was too high and reached 1.5% (S1 Table).

The characterization of microalgae strains by light microscopy showed that the dominant species in the Meishe river were *Chlorella*, *Tetradesmus*, *Tetraselmis*, *Cyclotella*, *Cryptomonas*, and *Scenedesmus*. The dominant species in the Shapo Reservoir consisted of *Scenedesmus*, *Microcystis*, *Oocystis*, *Planktothrix*, *Anabaena*, and *Gymnodinium*. The dominant species in Hongcheng Lake included *Phacus*, *Cryptomonas*, *Melosira*, *Gymnodinium*, and *Oscillatoria*.

*Chlorella* CHSA5 was cultured in 10 liters of water from the above-described water sources. *Chlorella* CHSA5 grew normally in the Meishe river and Shapo reservoir water samples. Moreover, algal cells reached peak levels on day 9 in the Meishe river water and on day 7 in the Shapo reservoir. The high salinity of the Hongcheng lake water negatively impacted *Chlorella* CHSA5 growth (S1 Fig). We chose water from the Meishe river for the simulated-field trial because the trial was located near the water collection point, and *Chlorella* was the dominant algal strain in the Meishe river.

**Fig 5:**
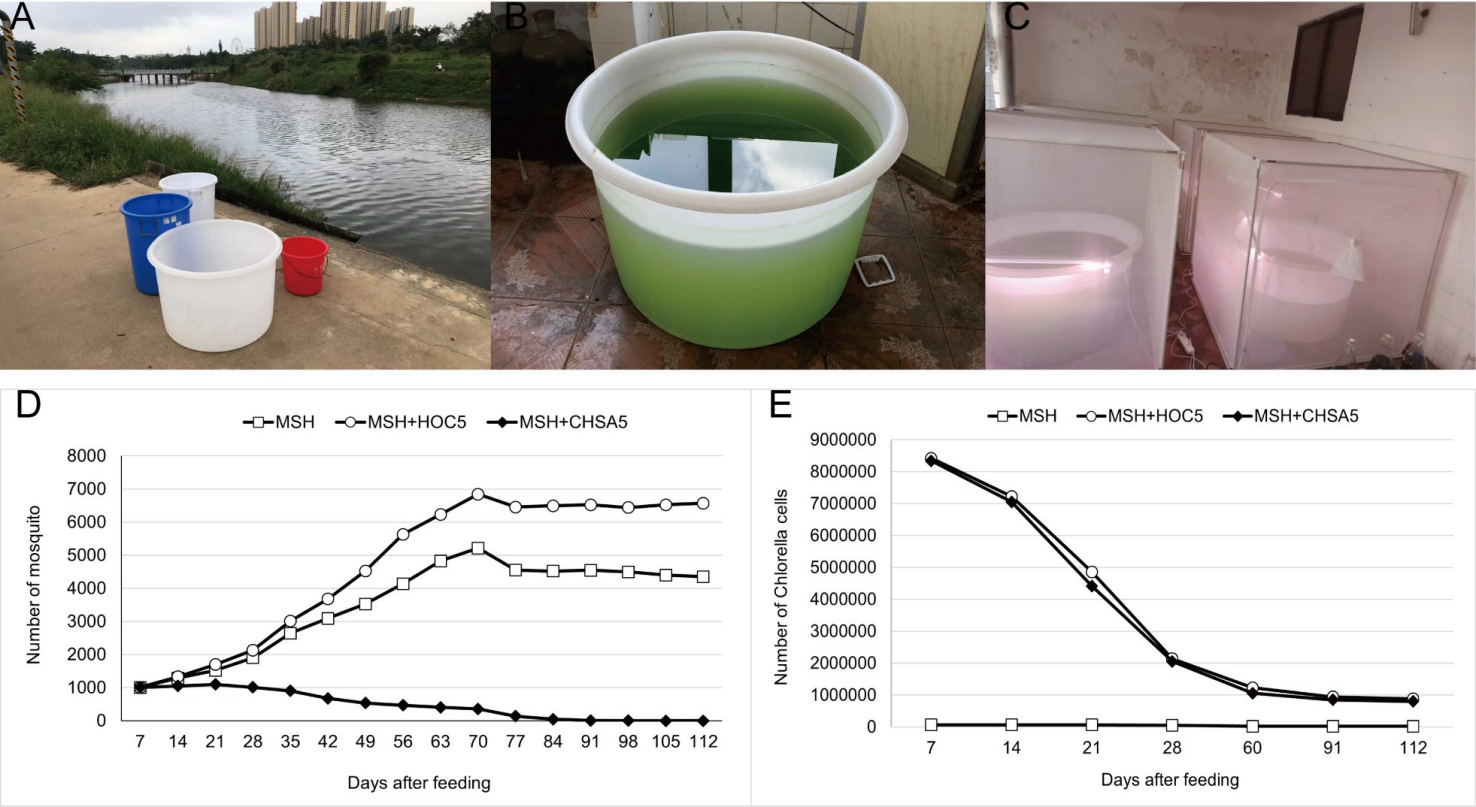
Simulated-field trials in suburban Haikou. The test water from Meishe river (A). 200 L *Chlorella* mixed with 600 L of the Meishe river water in the barrel (B). Approximately 1000 L1 larvae were placed in each cage, and the number of adult *Aedes* mosquitoes was counted once a week (C). Population and survival rates of *Ae*. *albopictus* in MSH, MSH+HOC5, and MSH+CHSA5 treatments (D). The relative abundance of *Chlorella* in MSH, MSH+HOC5, and MSH+CHSA5 waters (E). MSH: The mosquito was living in water from the Meishe river alone. MSH+HOC5: The mosquito was living in water from the Meishe river supplemented with wild Chlorella HOC5. MSH+CHSA5: The mosquito was living in water from the Meishe river supplemented with recombinant *Chlorella* CHSA5.

A simulated field release study was conducted to infer the potential environmental impacts of this technology. An idle factory building located 5 kilometers from the nearest residential area in the suburbs of Haikou City was selected for the experiment to reduce the impact of the trial on residents (Fig 5A, 5B and 5C). However, the experiment was performed using *Ae*. *Albopictus* instead of *Ae*. *aegypti* after considering the predominant dengue virus transmission in Hainan by *Ae*. *albopictus*. The number of *Aedes* mosquitoes fed the Meisher river water increased from 1000 to a maximum of 4825 individuals after 63 days and slowly decreased to approximately 4300 (112 days). The *Aedes* mosquitoes raised in the Meisher river water supplemented with wild-type *Chlorella* HOC5 increased to a maximum of 6844 individuals after 70 days and slowly decreased to approximately 6500 individuals (112 days). In the treatment group, *Aedes* mosquitoes raised in the Meisher river water supplemented with recombinant *Chlorella* CHSA5 decreased from 1000 to 0 (105 days) (Fig 5D).

The data indicate that *Chlorella* CHSA5 can suppress *Aedes* mosquito populations under simulated field conditions. However, the growth potential of *Chlorella* was limited, as the number of *Chlorella* in Meishe River was only 66900/mL. Generally, *Aedes* larvae feed on microalgae or microorganisms in the water. Adding *Chlorella* HOC5 significantly increased the number of *Aedes* mosquitoes in the Meishe river because *Chlorella* HOC5 increased the food density for *Aedes* (Fig 5E).

### Sequencing results of 18S V4 hypervariable region in the test water

An 18S high-throughput analysis was performed to understand the impact of recombinant *Chlorella* CHSA5 on biological populations of the test water. There were 85,340 tags qualified tags after quality control and 85,036 high-quality tags after removing unclassified and unique tags from the dataset. The total number of assigned OTUs was 2,021. The rarefaction curves of OTUs were prepared for all the samples, and each sample had a weak slope at the end of the curves (S2 Fig).

**Fig 6:**
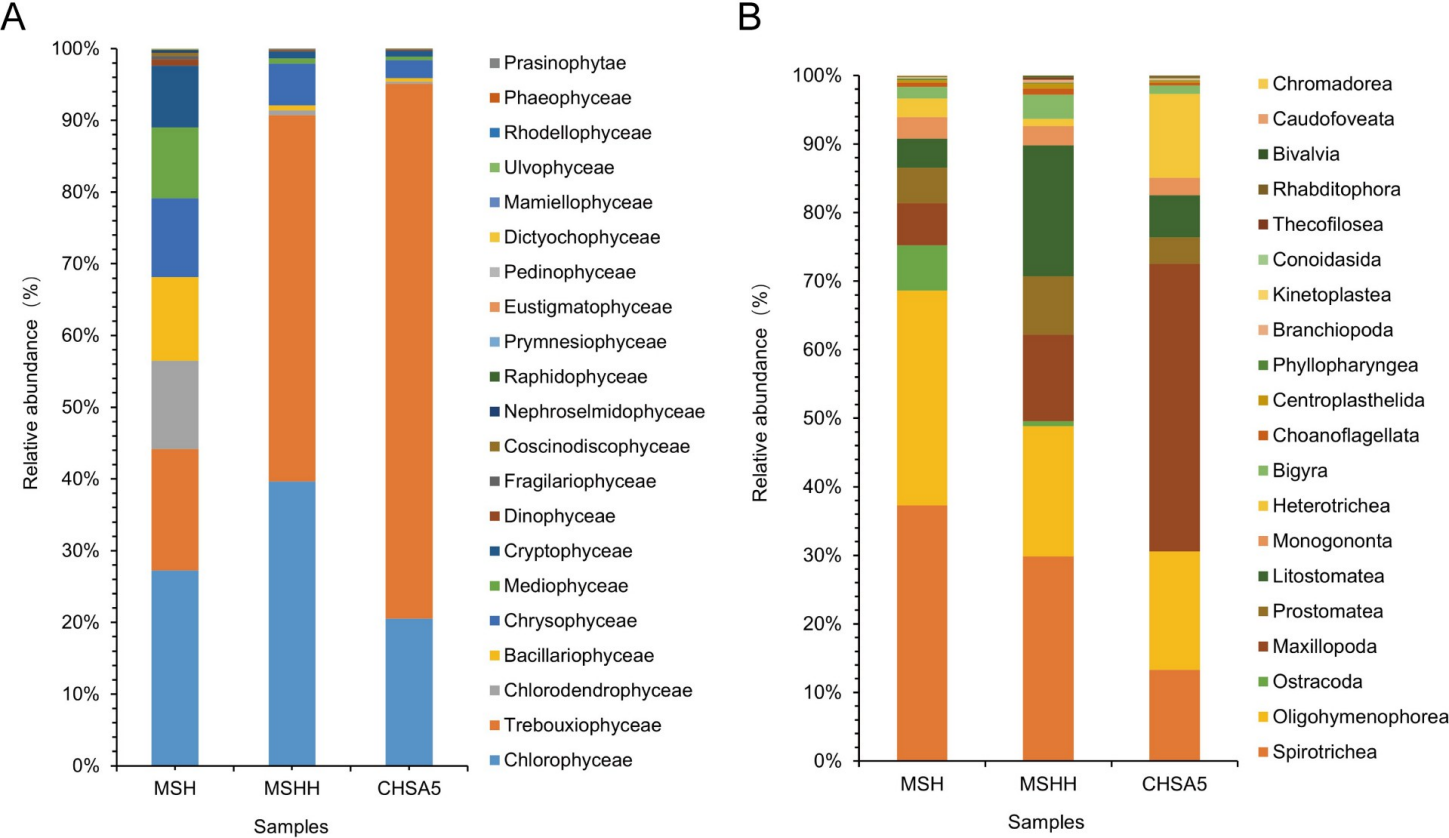
The relative abundance of phytoplankton (A) and zooplankton (B) at the class level in MSH, MSHH, and CHSA5 test waters. MSH: the mosquito was living in water from the Meishe river alone. MSHH: the mosquito was living in water from the Meishe river supplemented with wild *Chlorella* HOC5. CHSA5: the mosquito was living in water from the Meishe river supplemented with recombinant *Chlorella* CHSA5.

Twenty groups of eukaryotic microalgae were identified in the Meishe river (MSH) (at the class level), including *Chlorophycea*, *Chrysophyceae*, *Cryptophyceae*, and *Bacillariophyceae* (Fig 6A, S2 Table). The MSH groups contained 386 OTUs within 118 genera. *Chlorophycea* had the largest number of OTUs (75) within 32 genera. *Chrysophyceae*, the second largest class, contained 69 OTUs within 13 genera. *Cryptophyceae*, *Bacillariophyceae*, *Mediophyceae*, and *Dinophyceae* also hosted a relatively large number of OTUs (S2 Table). In contrast, *Phaeophyceae*, *Mamiellophyceae*, *Nephroselmidophyceae*, *Dictyochophyceae*, *Prymnesiophyceae*, *Raphidophyceae*, *Pedinophyceae*, *Ulvophyceae*, and *Rhodellophyceae* had much lower OTU numbers (S3 Fig, S2 Table).

The Meishe river *Chlorella* HOC5 group (MSHH) waters had 19 groups of microalgae at the class level (Fig 6A, S2 Table). Among them, 237 OTUs within 74 genera were observed in the MSHH group. *Chlorophycea* had the most OTUs (53) among 22 genera (Fig 6). *Chrysophyceae* was the second largest class with 44 OTUs among 6 genera. *Cryptophyceae*, *Bacillariophyceae*, *Dinophyceae*, *Trebouxiophyceae*, and *Mediophyceae* also hosted a relatively large number of OTUs (S2 Table). In contrast, *Pedinophyceae*, *Raphidophyceae*, *Rhodellophyceae*, *Mamiellophyceae*, *Prasinophytae*, *Chlorodendrophyceae*, *Fragilariophyceae*, *Ulvophyceae*, *Nephroselmidophyceae*, *Phaeophyceae* and *Prymnesiophyceae* had much lower OTU numbers (S3 Fig, S2 Table).

In the CHSA5 treatment, 16 eukaryotic microalgae were identified at the class level from the Meishe river and recombinant *Chlorella* CHSA5 waters (CHSA5). These included *Chlorophyceae*, *Chrysophyceae*, *Cryptophyceae*, and *Bacillariophyceae* (Fig 6A). Of these, 206 OTUs within 67 genera were observed in the CHSA5 group. *Chlorophyceae* had the largest number of OTUs (46) among the 18 genera of *Chlorophyceae* in the CHSA5 group. *Chrysophyceae* was the second largest class with 37 OTUs across 8 genera. *Trebouxiophyceae*, *Bacillariophyceae*, *Dinophyceae*, *Cryptophyceae*, and *Mediophyceae* also hosted a large number of OTUs (S2 Table). In contrast, *Fragilariophyceae*, *Prymnesiophyceae*, *Ulvophyceae*, *Chlorodendrophyceae*, *Raphidophyceae*, *Phaeophyceae*, *Nephroselmidophyceae* and *Eustigmatophyceae* had much lower OTU numbers (S3 Fig, S2 Table).

### Variation of phytoplankton community in the test water

**Fig 7:**
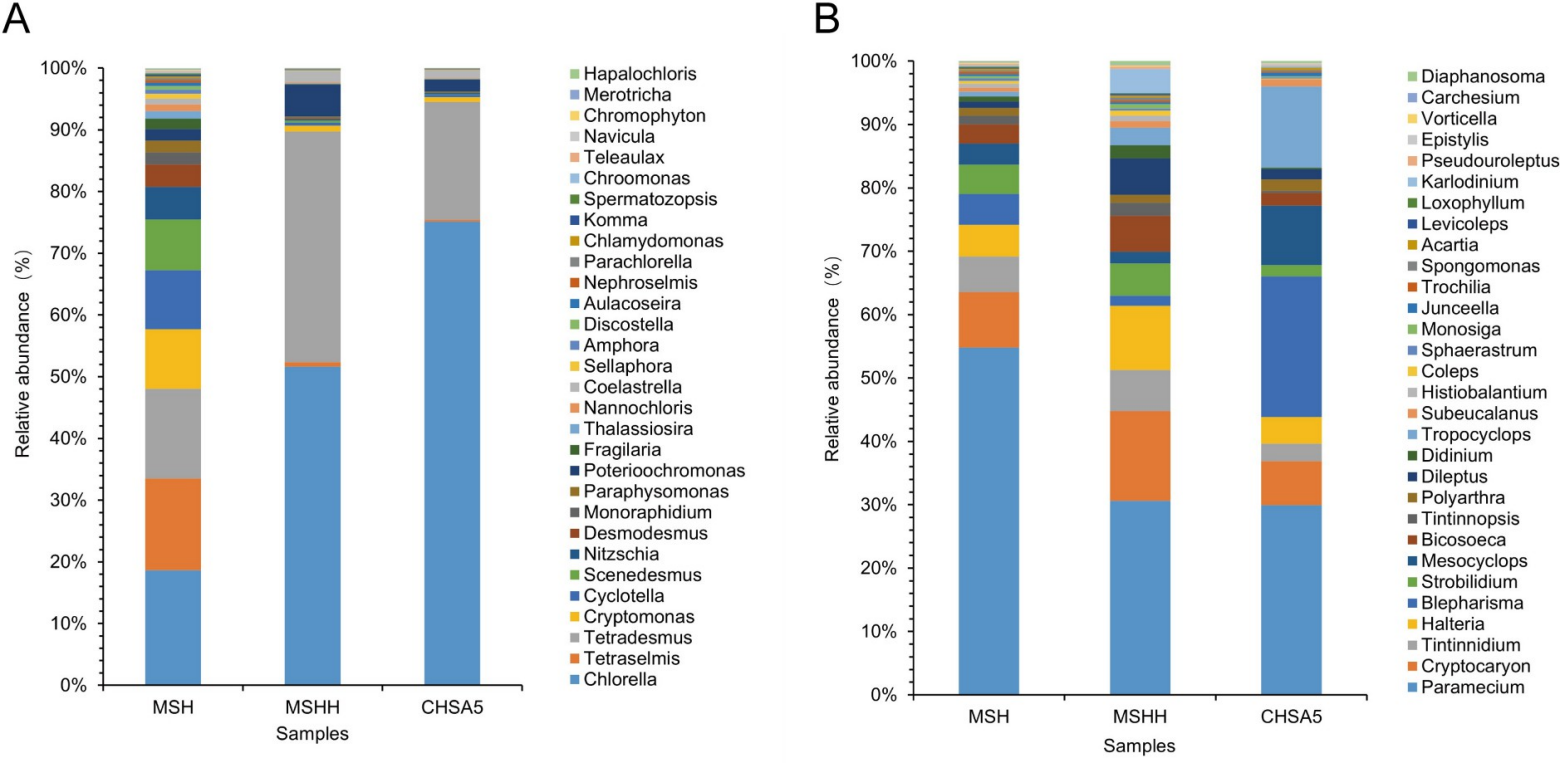
The relative abundance of phytoplankton (A) and zooplankton (B) at the genus level in MSH, MSHH, and CHSA5 test waters. MSH: the mosquito was living in water from the Meishe river alone. MSHH: the mosquito was living in water from the Meishe river supplemented with wild *Chlorella* HOC5. CHSA5: the mosquito was living in water from the Meishe river supplemented with recombinant *Chlorella* CHSA5.

**Fig 8:**
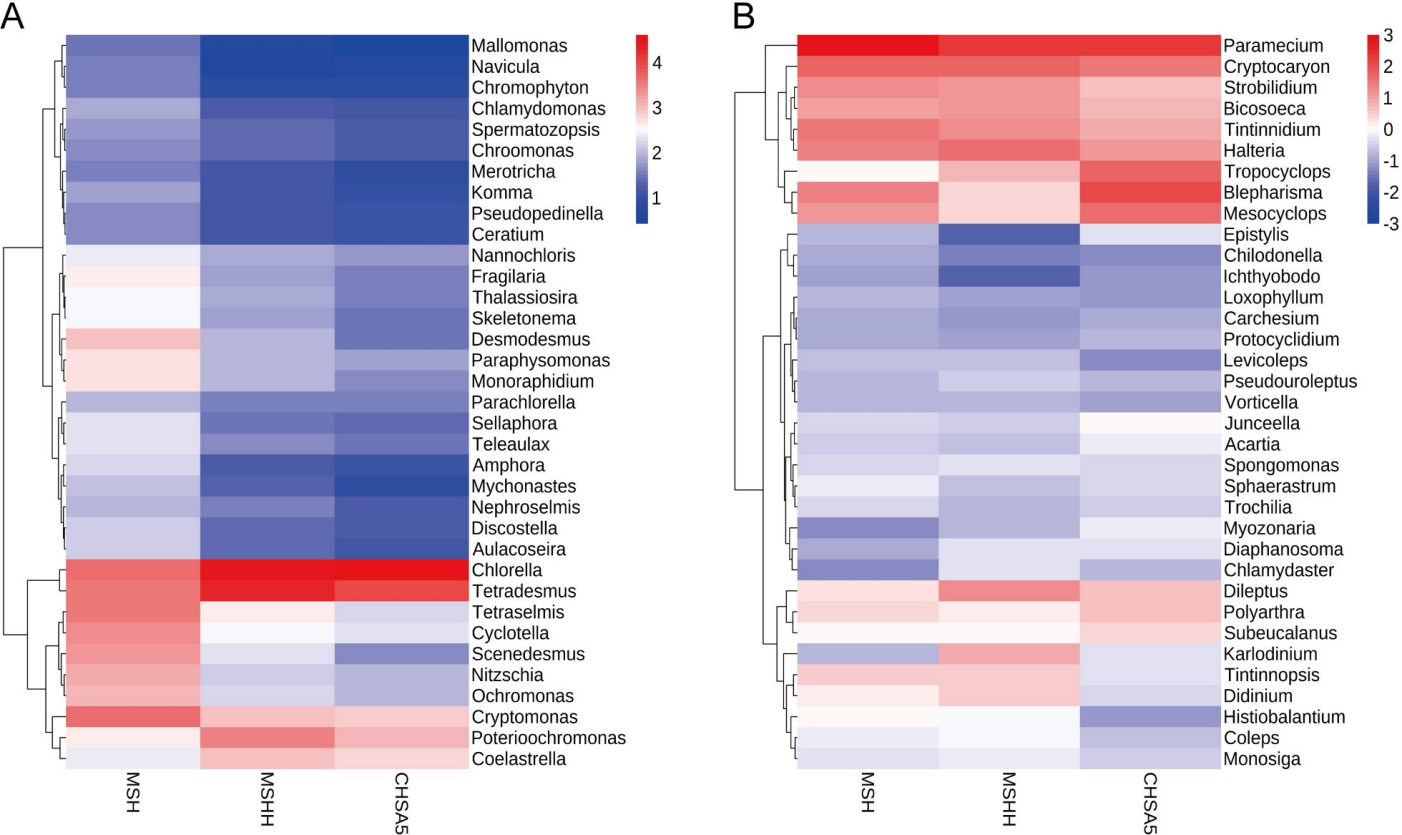
Heat maps showing the phytoplankton (A) and zooplankton (B) in the MSH, MSHH, and CHSA5 test waters. The horizontal coordinate and the right and left sides indicate the sample names and classes of phytoplankton or zooplankton, respectively. The color of the heat map is negative when the values are below the mean and positive when above. Values of the heat maps, as indicated by the color scale at the top right corner, are calculated as the standard score (Z-values) z = (x-μ)/σ, where: x is the relative abundance of a specific group of phytoplankton. μ is the mean of the relative abundance of all different groups of phytoplankton. σ is the standard deviation of the relative abundance of all different groups of phytoplankton. The similarity metric was calculated by Bray–Curtis distance for hierarchical clustering using R software. MSH: the mosquito was living in water from the Meishe river only. MSHH: the mosquito was living with water from the Meishe river supplemented with wild *Chlorella* HOC5. CHSA5: the mosquito was living in waters from the Meishe river supplemented with recombinant *Chlorella* CHSA5.

**Fig 9:**
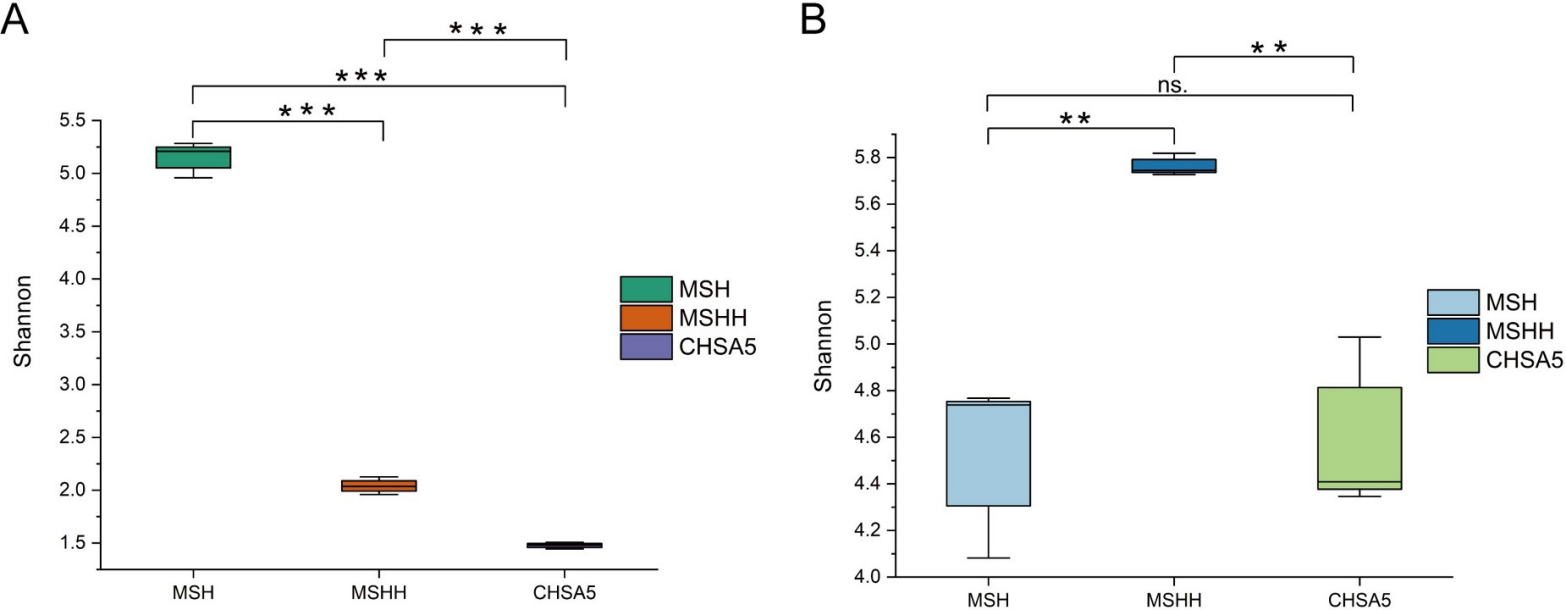
Shannon diversity indexes of phytoplankton (A) and zooplankton (B) in the test waters of MSH, MSHH, and CHSA5. MSH: the mosquito was living in the water from the Meishe river alone. MSHH: the mosquito was living in water from the Meishe river supplemented with wild *Chlorella* HOC5. CHSA5: the mosquito was living in water from the Meishe river supplemented with recombinant *Chlorella* CHSA5.

The MSH group had the highest species diversity. Adding excess *Chlorella* HOC5 to the Meishe river water increased the relative abundance of *Chlorella* from 18.6 to 51.6% (MSHH group). In the CHSA5 treatment group, the relative abundance of *Chlorella* rose to 75.2%. Moreover, the number of phytoplankton in the MSHH group decreased by 44 at the genus level and 149 OTUs due to the rapid increase of *Chlorella* relative to the dynamics observed in the MSH group. The number of phytoplankton in the CHSA5 group decreased by 51 at the genus level and 180 OTUs (Fig 7A, S3 Fig, S4 Fig, S2 Table). The relative abundance of other major species decreased in the MSHH and CHSA5 groups compared to the MSH group, except for *Chlorella*, *Tetradesmus*, *Poterioochromonas*, and *Coelastrella* (Figs 7A and 8A). Furthermore, the MSH group had a higher Shannon diversity index (5.15) than the MSHH (2.04) and CHSA5 groups (1.48). The Shannon index of the MSH group was significantly different from that of the MSHH and CHSA5 groups (P ≤0.01). There was also a significant difference in the Shannon indexes between the MSHH and CHSA5 groups (P ≤0.01) (Fig 9A). The above results show that rapidly increasing the wild-type or recombinant *Chlorella* is related to the death and disappearance of other microalgae. Since the relative abundance of recombinant *Chlorella* in the CHSA5 group was higher than that of wild-type *Chlorella* in the MSHH group, the species decreased the most, and the diversity index was the lowest in the CHSA5 group.

### Variation of zooplankton community in the test water

A total of 29, 37, and 29 groups of protozoa were identified at the class level in the MSH, MSHH, and CHSA5 groups, respectively. *Spirotrichea*, *Oligohymenophorea*, *Maxillopoda*, *Prostomatea*, *Litostomatea*, *Bigyra*, and *Choanoflagellata* were the majority of the observed protozoans (Fig 6B, S3 Table). Among these, there were 207 OTUs among 53 genera in the MSH group, 239 OTUs within 54 genera in the MSHH group, and 201 OTUs within 50 genera in the CHSA5 group (S3 Table).

In contrast to the zooplankton species in the MSH group, the number of zooplankton species increased by 1 genus and 32 OTUs when wild-type *Chlorella* HOC5 was added to the MSHH group, while the zooplankton species in the CHSA5 group decreased by 3 genera and 6 OTUs (Fig 7B, S3 Table). In the CHSA5 group, the relative abundance of the main genera (*Paramecium*, *Cryptocaryon*, *Strobilidium*, *Bicosoeca*, *Tintinnidium*, and *Halteria)* decreased compared to the MSH and MSHH groups. In contrast, the relative abundance of *Tropocyclops*, *Blepharisma*, *Mesocyclops*, and *Epistylis* increased (Figs 7B and 8B). The Shannon diversity indexes were 4.52, 5.76, and 4.59 in the MSH, MSHH, and CHSA5 groups, respectively. The Shannon index of the MSHH group was significantly higher than that of the MSH and CHSA5 groups (P ≤0.01) but not significantly different between the MSH and CHSA5 groups (Fig 9B). These results indicate that adding wild-type *Chlorella* to the MSHH group result in algal blooms and increase the number of zooplankton species feeding on *Chlorella*. However, adding recombinant *Chlorella* to the CHSA5 group slightly decreased the number of zooplankton species, probably due to the *chsa* dsRNA fragment carried by the recombinant *Chlorella*. Since chitin is present on protozoan surface coats [48], the *chsa* dsRNA in recombinant *Chlorella* possibly inhibited the expression level of *chsa* in protozoa, thus, decreasing the number of protozoan species in the CHSA5-treated water.

## Discussion

*Aedes* mosquitoes are vectors of epidemic diseases that affect public health worldwide, such as yellow fever, dengue fever, Zika virus, and chikungunya virus [49–52]. These diseases cause a large number of deaths every year. Without effective therapies or vaccines, vector control is the most effective strategy to prevent the spread of these diseases. Numerous studies have shown that RNAi is a powerful tool for controlling insect populations and may be less susceptible to insect resistance than other strategies. In this study, *Chlorella*, a natural food for mosquito larvae, served as the exogenous RNAi carrier. Recombinant *Chlorella* can be directly released into suburban waters as a biological pesticide. This biological "insecticide" can self-replicate, lowering production costs.

In this study, the *Aedes* mosquito *chsa* gene was used as the target for RNAi. Since this gene primarily exists in invertebrates and fungi, it is likely to have a limited impact on humans, higher animals, and plants. However, environmental impacts are unclear [48–57]. *Chlorella*, a dominant algal strain widely in local waters, was selected as the host for genetic manipulation. The genetically modified mosquito-killing *Chlorella* adapted to environmental conditions, allowing for rapid growth to reach a desired level of relative abundance. Some of the *Ae*. *aegypti* larvae fed on recombinant *Chlorella* died the next day, and all the larvae died within 13 days (Fig 1A). In the feeding experiment of 300 *Aedes* mosquitoes, 88.5% of the larvae fed on recombinant *Chlorella* died within 30 days. These results indicate that oral administration of *chsa* RNAi recombinant *Chlorella* is lethal to *Ae*. *aegypti* larvae (Fig 2A). Subsequently, *Ae*. *albopictus* were fed the recombinant *Chlorella*. The results showed that the recombinant *Chlorella* had a lethal effect on *Ae*. *albopictus* (Fig 3A and 3B). The 290 bp target sequence homology of *Ae*. *aegypti* and *Ae*. *albopictus chsa* coding region sequence was 91.3%, which was expected.

Considering that the main vector of the dengue virus (*Ae*. *Albopictus*) in Hainan, China, this species was used in the simulated field experiment study [58,59]. The waters of the Meishe river in Haikou City were used for seeding using recombinant *Chlorella*, one of the predominant microalgae strains in the local ecosystem. In addition, the eutrophication of the Meishe river, with excessive N, P, ammonia, and COD (S1 Table), was favorable for the survival and reproduction of the recombinant *Chlorella* (S1 Fig).

The test results showed that the recombinant *Chlorella* reduced the number of *Aedes* mosquitoes from 1000 to 0 within 105 days. The *Ae*. *albopictus* population was effectively suppressed (Fig 5D). 18S high-throughput sequencing analysis was performed to understand the impact of recombinant *Chlorella* on biological populations in the test water. Adding excess recombinant *Chlorella* to the Meishe River increased its relative abundance by 76.6%. However, it decreased the number of phytoplankton by 51 (at the genus level) and the number of OTUs by 179 (Fig 7, S3 and S4 Figs). This outcome is the result of nutritional competition. The rapid reproduction of *Chlorella*, similar to the phenomenon of algal blooms in natural waters, where *Chlorella* primarily consumed nutrients in the water, resulted in the death of a portion of the naturally occurring phytoplankton (Fig 7A, Fig 8A). The proliferation of *Chlorella* has also led to an increase in the number of zooplankton species that feed on wild *Chlorella*. However, the *chsa* dsRNA carried by the recombinant *Chlorella* may inhibit the expression of *chsa* genes in several species of protozoans with high homology, resulting in a decrease in the number of protozoan species (Fig 7B, Fig 8B) in the CHSA5 group.

Although some progress has been made in this study, the impact of exogenous DNA fragments carried by recombinant *Chlorella*, especially antibiotic resistance genes, on environmental organisms, including the organisms in direct and indirect food chains, needs to be further evaluated. The research aimed to develop low-cost, environmentally friendly mosquito control technologies. Although this self-propagating microalgae insecticide has been validated in the laboratory, further assessment of the effects of blooms caused by recombinant *Chlorella* after release into waters is necessary. There have been many reports on harmful algal blooms [60–62] mainly caused by microalgae-containing toxins. These toxin-containing microalgae, including *Alexandrium* species, *Gymnodinium catenatum*, *Prorocentrum lima*, *Dinophysis acuta*, *Anabaena*, and *Cylindrotheca*, can be transmitted to mammals and humans through fish and shellfish, threatening human health [63]. Non-toxic algal blooms have little impact on ecosystems and are rarely studied and discussed. Non-toxic recombinant *Chlorella* should be kept at levels that can control mosquito populations and have little impact on other plankton species, making it acceptable to the public. While promising, this approach will require refinement, including *in vitro* gene editing technology, shRNAi technology, etc., to minimize the impact of exogenous DNA fragments carried by recombinant microalgae so that the technology can be employed with a high degree of safety.

## Supporting information

S1 Fig The growth curve of *Chlorella* CHSA5 in the waters from Shapo reservoir, Hongcheng lake, and Meishe river. (TIF)

S2 Fig Rarefaction curves of OTUs for all samples collected from the test waters. Propinquity to saturation is denoted by weak slopes at the end of rarefaction curves. Sequences with ≥97% similarity were assigned to an OTU. MSH1-3: the mosquito was living in water from the Meishe river alone. MSHH1-3: the mosquito was living in water from the Meishe river supplemented with wild *Chlorella* HOC5. CHSA1-3: the mosquito was living in water from the Meishe river supplemented with recombinant *Chlorella* CHSA5.
(TIF)

S3 Fig The richness of genera (A, C, and E) and OTUs (B, D, and F) within different groups of MSH, MSHH, and CHSA5. The unidentified taxa are OTUs that were not assigned to any known groups. MSH: the mosquito was living in water from the Meishe river alone. MSHH: the mosquito was living in water from the Meishe river supplemented with wild *Chlorella* HOC5. CHSA5: the mosquito was living in water from the Meishe river supplemented with recombinant *Chlorella* CHSA5.
(TIF)

S4 Fig The top 20 microalgal genera were identified in MSH (A), MSHH (B), and CHSA5(C) test waters. *Chlorella* is labeled using the underscore. MSH: the mosquito was living in water from the Meishe river alone. MSHH: the mosquito was living in water from the Meishe river supplemented with wild *Chlorella* HOC5. CHSA5: the mosquito was living in water from the Meishe river supplemented with recombinant *Chlorella* CHSA5.
(TIF)

S1 Table Water quality detection of the Meishe river, Shapo reservoir, and Hongcheng lake. (TIF)

S2 Table Genus and OTU numbers of the phytoplankton within different groups of MSH, MSHH, and CHSA5 at the class level. (TIF)

S3 Table Genus and OTU numbers of the zooplankton within different groups of MSH, MSHH, and CHSA5 at the class level. (TIF)
