# Supplementary figures and images for: Control of Aedes mosquito populations using recombinant microalgae expressing short hairpin RNAs and their effect on plankton
Source: PLoS Negl Trop Dis. 2023 Jan 26;17(1):e0011109. doi: 10.1371/journal.pntd.0011109 (PMC9904476; doi:10.1371/journal.pntd.0011109)

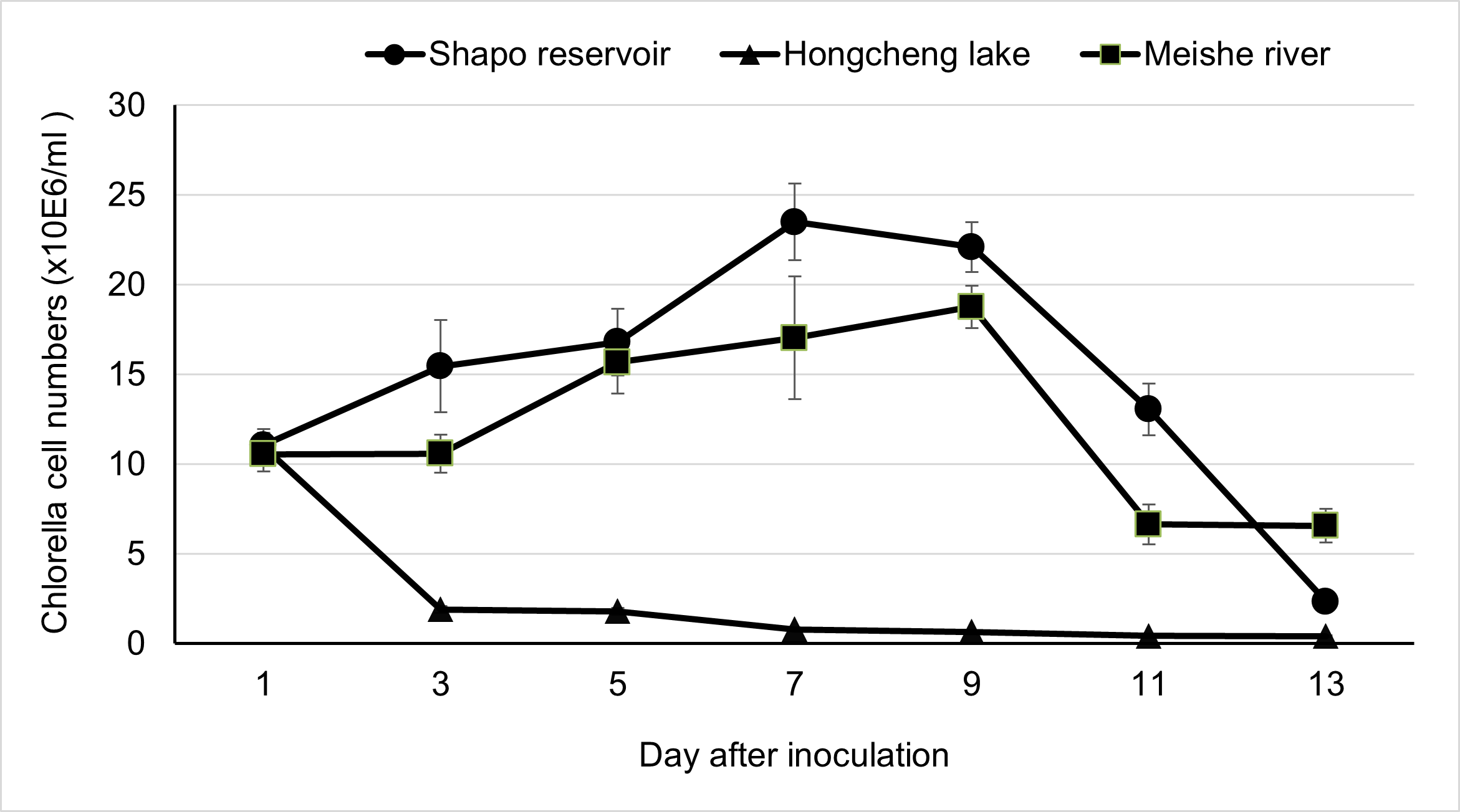

Supplement: S1 Fig — (TIF) [file pntd.0011109.s001.tif]

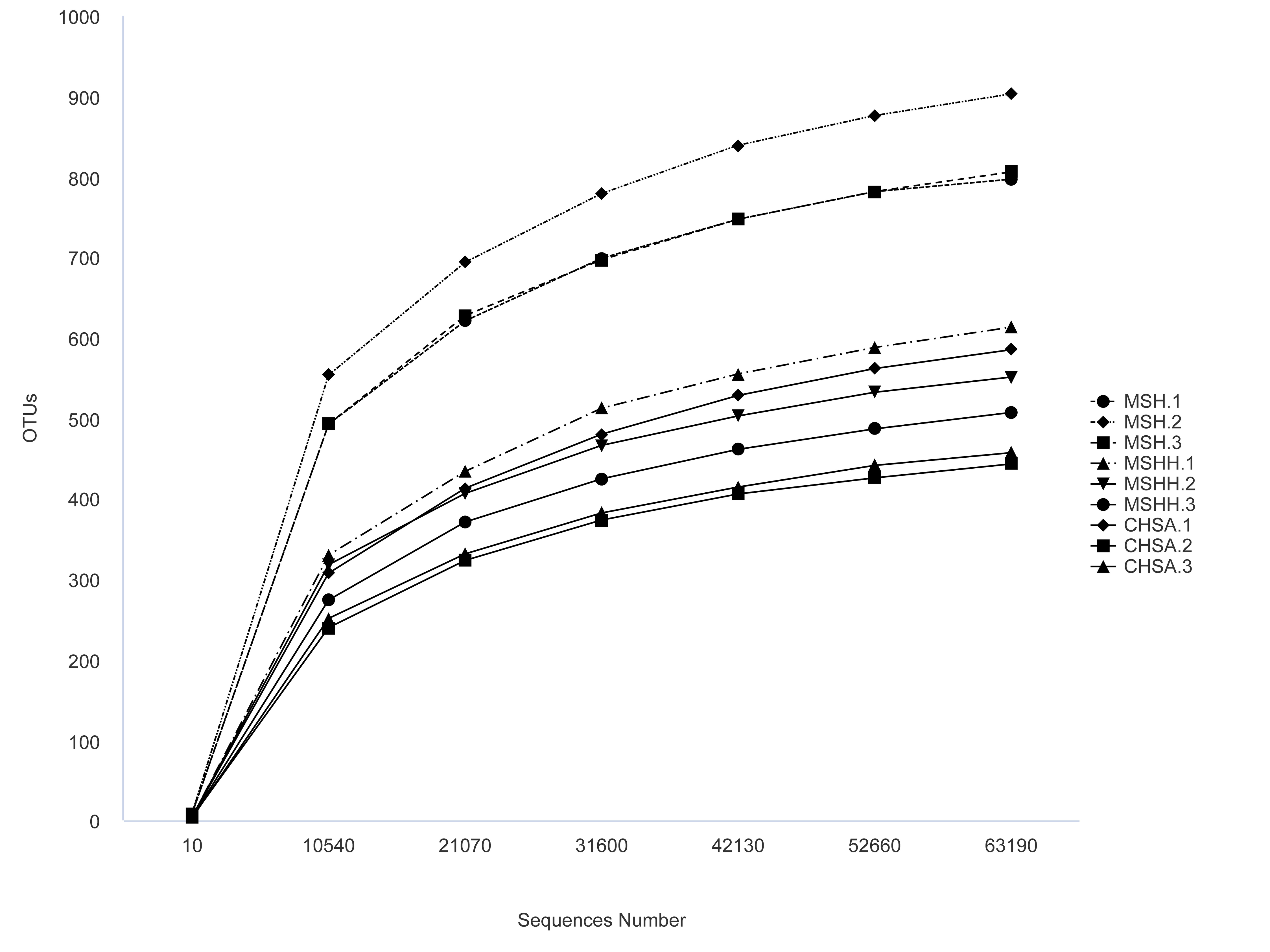

Supplement: S2 Fig — Propinquity to saturation is denoted by weak slopes at the end of rarefaction curves. Sequences with ≥97% similarity were assigned to an OTU. MSH1-3: the mosquito was living in water from the Meishe river alone. MSHH1-3: the mosquito was living in water from the Meishe river supplemented with wild Chlorella HOC5. CHSA1-3: the mosquito was living in water from the Meishe river supplemented with recombinant Chlorella CHSA5. (TIF) [file pntd.0011109.s002.tif]

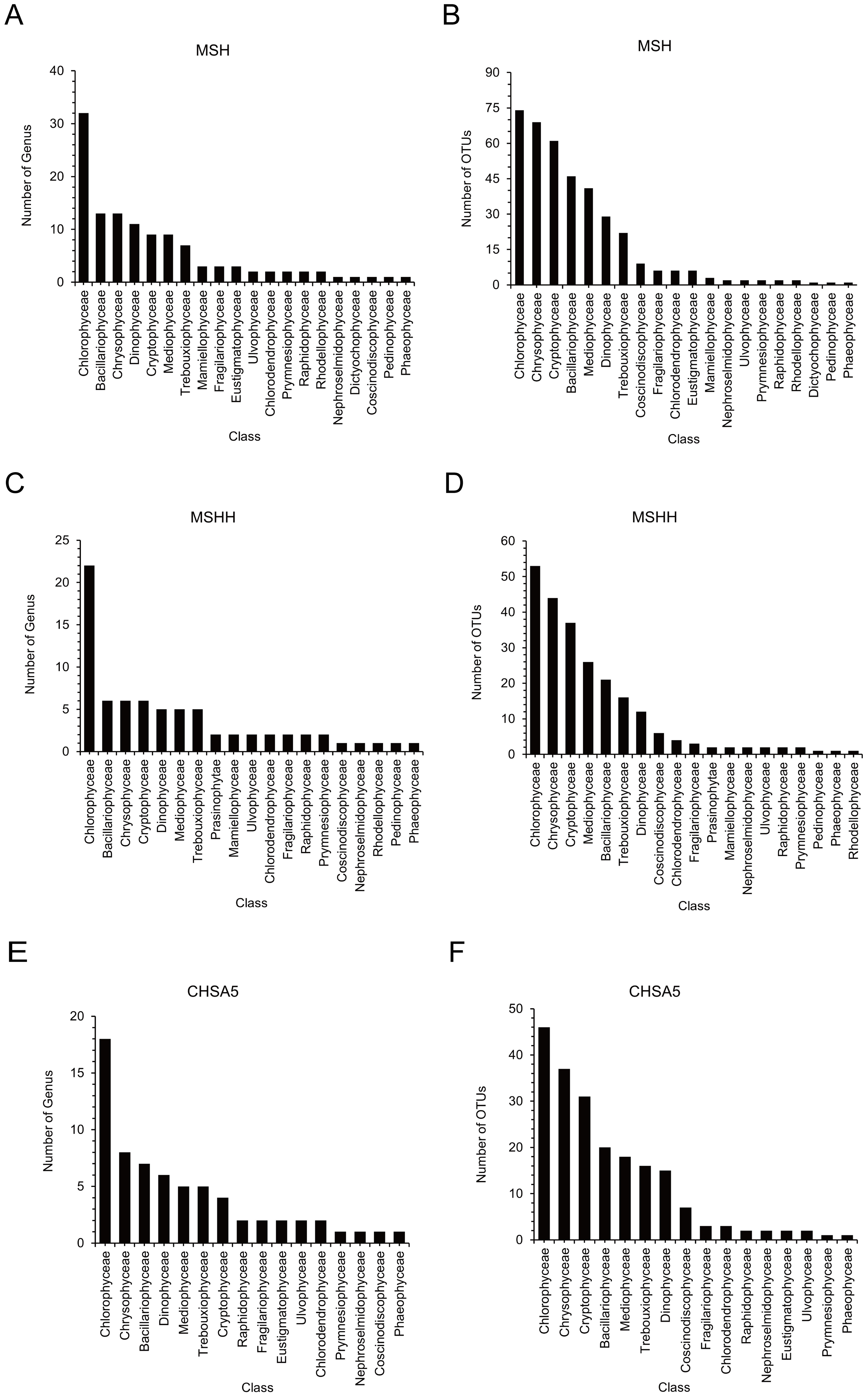

Supplement: S3 Fig — The richness of genera (A, C, and E) and OTUs (B, D, and F) within different groups of MSH, MSHH, and CHSA5. The unidentified taxa are OTUs that were not assigned to any known groups. MSH: the mosquito was living in water from the Meishe river alone. MSHH: the mosquito was living in water from the Meishe river supplemented with wild Chlorella HOC5. CHSA5: the mosquito was living in water from the Meishe river supplemented with recombinant Chlorella CHSA5. (TIF) [file pntd.0011109.s003.tif]

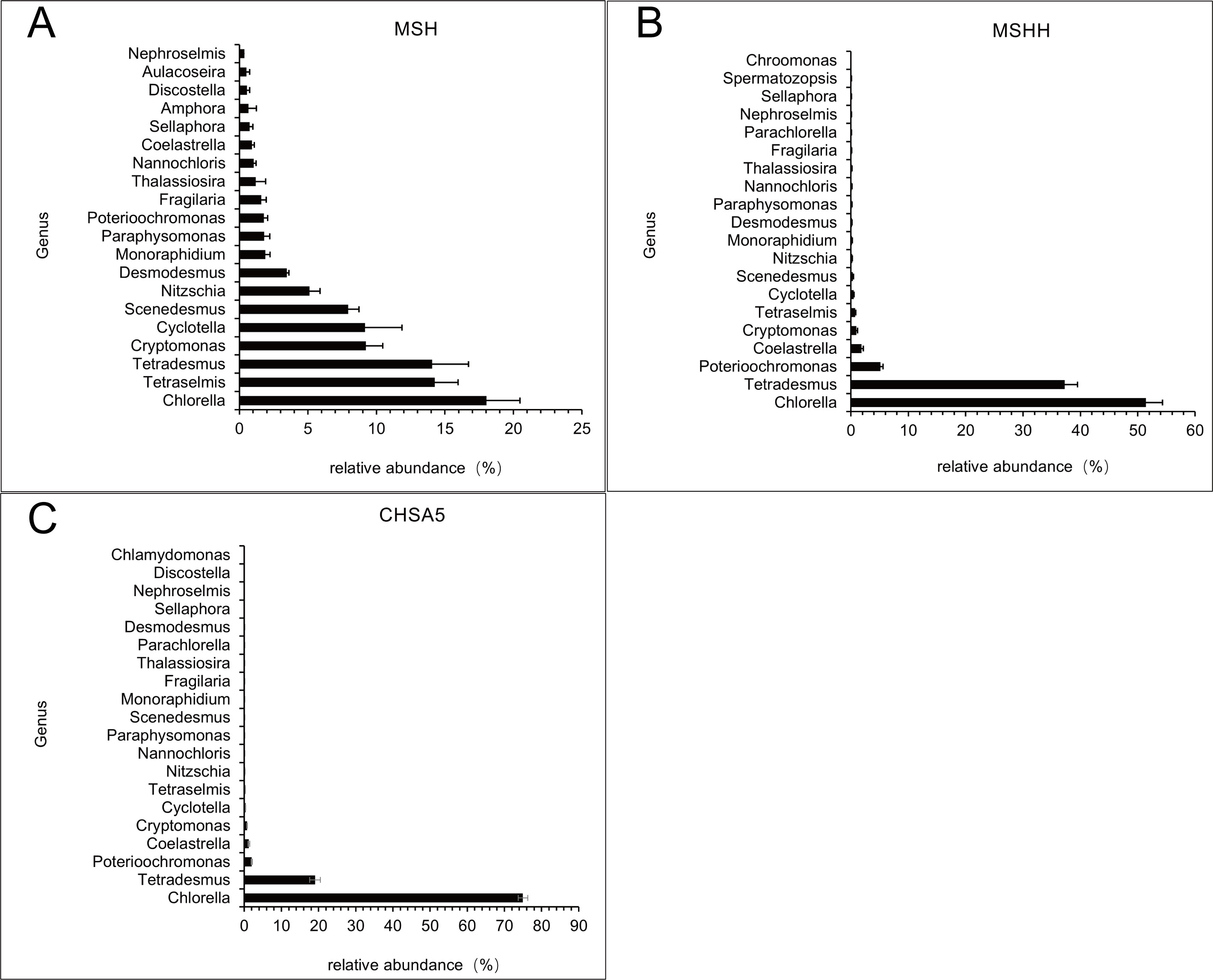

Supplement: S4 Fig — The top 20 microalgal genera were identified in MSH (A), MSHH (B), and CHSA5(C) test waters. Chlorella is labeled using the underscore. MSH: the mosquito was living in water from the Meishe river alone. MSHH: the mosquito was living in water from the Meishe river supplemented with wild Chlorella HOC5. CHSA5: the mosquito was living in water from the Meishe river supplemented with recombinant Chlorella CHSA5. (TIF) [file pntd.0011109.s004.tif]

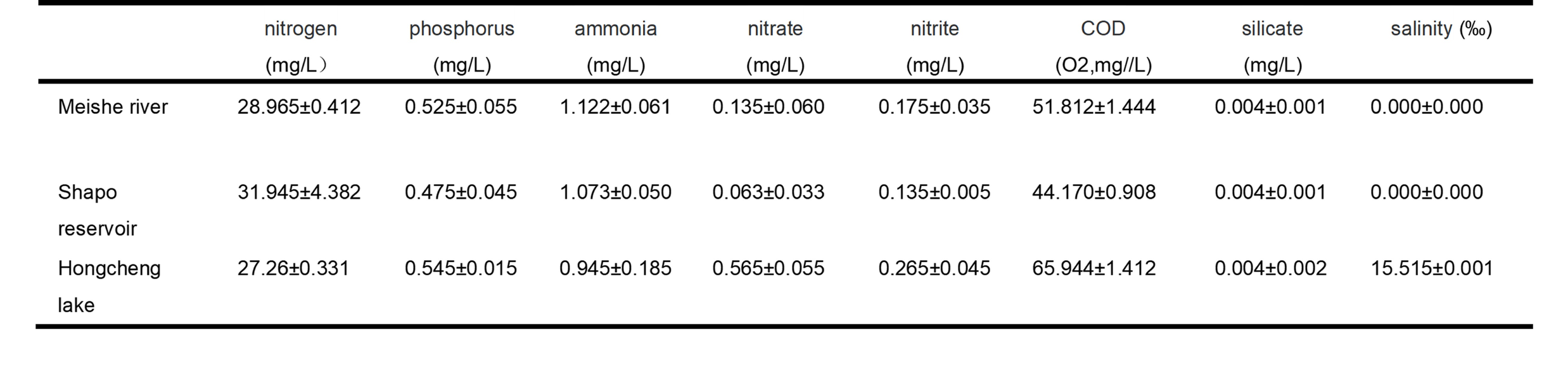

Supplement: S1 Table — (TIF) [file pntd.0011109.s005.tif]

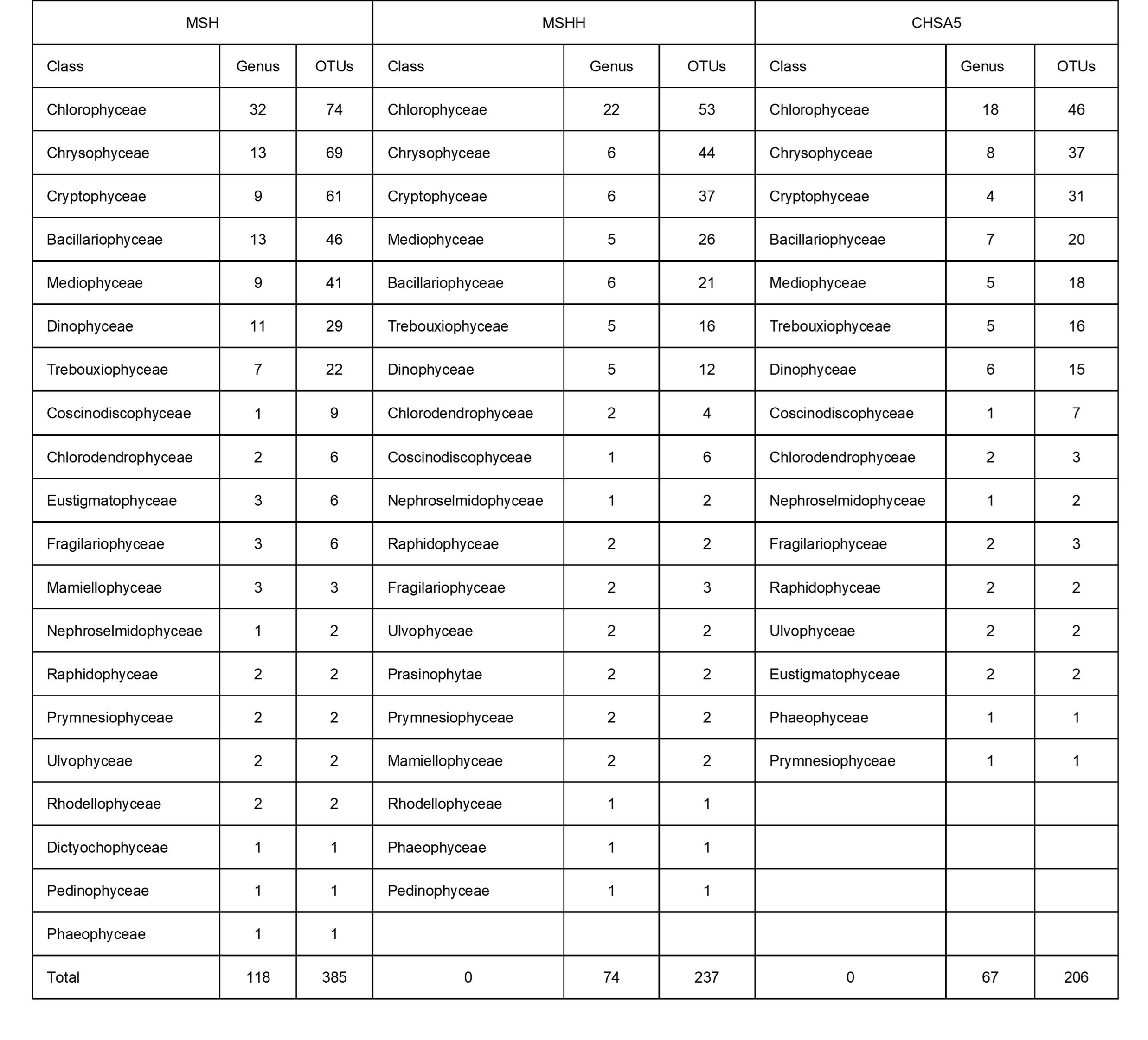

Supplement: S2 Table — (TIF) [file pntd.0011109.s006.tif]

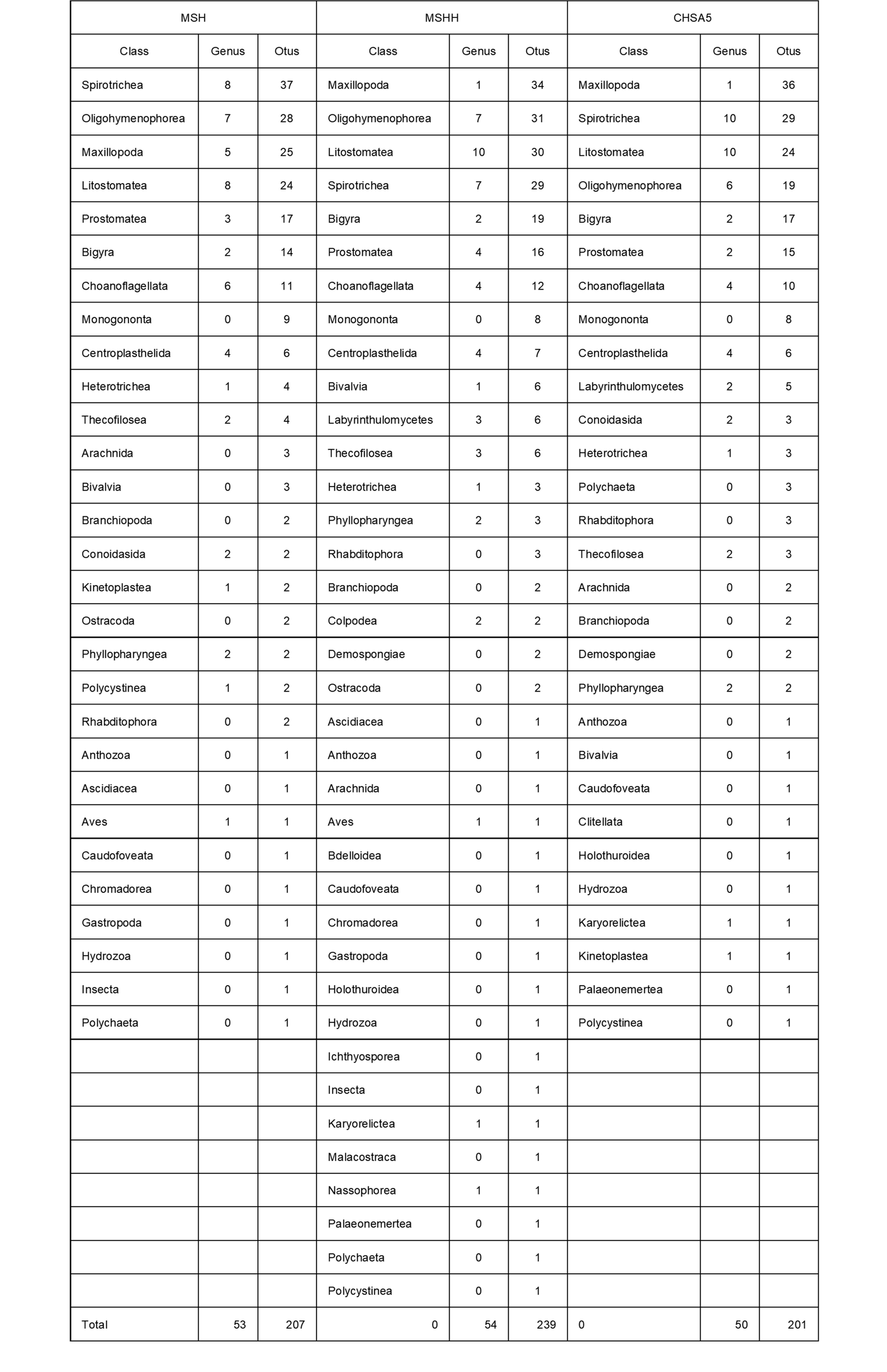

Supplement: S3 Table — (TIF) [file pntd.0011109.s007.tif]
